# Investigation of antibiosis, anti-diabetic, antioxidant, anti-inflammatory, molecular docking and dye degradation potential of green synthesized copper ferrite (CuFe_2_O_4_) nanoparticles using mushroom *Pleurotus florida*

**DOI:** 10.1186/s11671-025-04251-5

**Published:** 2025-06-30

**Authors:** Beena Cherian, Tijo Cherian, Teena Merlin, Shilpa Jose

**Affiliations:** 1 Department of Food Technology and Quality Assurance, School of Biosciences, Mar Athanasios College for Advanced Studies (MACFAST), Tiruvalla, Kerala 689101 India; 2 Department of Marine Biology and Ocean Studies, Pondicherry University, Port Blair Campus, Andamans, 744112 India; 3 School of Biosciences, Mar Athanasios College for Advanced Studies (MACFAST), Tiruvalla, Kerala 689101 India; 4 St. Teresa’s College, Ernakulam, Kerala 682011 India

**Keywords:** Antibacterial, Anti-diabetic, Antioxidant, Anti-inflammatory, Molecular docking, Copper ferrite nanoparticles, Dye degradation, *Pleurotus florida*

## Abstract

The current study proposes a low-cost, environmentally benign manufacturing approach of copper ferrite nanoparticles (CuFe_2_O_4_ NPs) via mushroom extract of *Pleurotus florida* (PFE) as the first-time report. Several characterization methods verified the production of PFE-CuFe_2_O_4_ NPs. The absorption spectrum exhibited the peak at 420 nm; band gap of 1.85 eV. The studies of SEM and TEM confirmed spherical and homogeneously distributed CuFe_2_O_4_ NPs with an average size of 22.4 ± 1.4 nm. The FTIR reported the presence of bio-essential molecules in PFE can act as a stabilizing and capping agent. The NPs were found to be fairly stable with zeta potential found at 28.9 ± 0.2 mV. Numerous in vitro biological investigations exemplified the applicability and practicality of CuFe_2_O_4_ NPs and compare them with the standard. The biofunctionalized CuFe_2_O_4_ NPs demonstrated a potent antibacterial activity against *E. coli* and *S*. *aureus*. Additionally, it was discovered that CuFe_2_O_4_ NPs have superior antioxidant activity (77–83%) and their scavenging ability is more comparable to ascorbic acid (control). Furthermore, a degradation efficiency of 91–92% was observed in 10–15 min for CuFe_2_O_4_ NPs in rhodamine B (RhB) and methylene blue (MB) dyes, indicating their remarkable effectiveness in this regard. Future research may focus on applying CuFe_2_O_4_ NPs to comprehensive wastewater treatment and determining the degradation products and ecological consequences.

**Supplementary Information:**

The online version contains supplementary material available at 10.1186/s11671-025-04251-5.

## Introduction

The domain of biotechnology has greatly benefited from the wide openings provided by nanotechnology, which has produced important discoveries with implications for a wide range of applications. Because of their tunable mechanical, chemical, and physical characteristics and superior performance over their bulkier equivalents, nanoparticles (NPs) have become increasingly popular in technological applications [1]. Nanoparticles have emerged as a subject of considerable curiosity, given their wide range of uses in industries like electronics, cosmetics, drug delivery systems, biosensors, agriculture, pharmaceutics, health, biology, optics, and catalysis [2–5]. Different physical and chemical techniques are used to create these NPs; however, these methods have certain disadvantages, such as high expenditures, usage of high pressures, temperatures and harmful chemicals that poses perilous threat to biotic life and environment [6, 7]. As a result, researchers are now using biological approaches due to the advent of workable green synthesis techniques with more focus placed on recent advancements in nanobiotechnology, green synthesis and the environmentally beneficial creation of nanoparticles from living organisms [8, 9]. A bottom-up technique called"green synthesis"produces metal nanoparticles and their oxides by using a natural extract from a product—like fruit or leaves from a tree or crop—instead of a costly chemical reducing agent. Biological species offer a huge potential for producing NPs. Aside from being economical, environmentally benign, sustainable, and free of chemical contamination, the biological reduction of metal initiators to match NPs is also appropriate for mass production. Furthermore, the green manufacturing of nanoparticles makes it possible to recycle exorbitant metal salts like gold and silver in waste streams [1–9]. Green biosynthesis procedures are quick, simple, inexpensive, proficient, and eco-beneficial compared to chemical and physical methods [10]. In actuality, the components of the extract serve as possible reducers and capping agents, restricting the excessive growth and aggregation of nanoparticles during colloidal formation. These compounds could improve the efficacy and applications of the resulting nanoparticles by influencing and enhancing their characteristics [6]. The nanoparticles produced using this environmentally friendly process offer special physical and chemical features, environmental and economic advantages, such as readily available, low synthetic expenditures, safety, and no toxic effects. Biological qualities are greatly enhanced with anti-cancer, anti-oxidant, anti-microbial, and increased biocompatibility characteristics. These exceptional qualities enable more research into their potential uses in agriculture and medicine [1]. A comparative account of traditional and green approaches employed in the synthesis of copper ferrite nanoparticles has been mentioned in Table 1.

**Table 1:** Traditional and green approaches for the synthesis of CuFe_2_O_4_ nanoparticles

Composition of nanoparticles	Method of preparation	Type of approach	References
Cu_1-x_M_x_Fe_2_O_4_	Thermal decomposition	Traditional	[31]
CuCe_x_Fe_2-x_O_4_	Molten-Salt	Traditional	[32]
In-Cu ferrite	Co-precipitation	Traditional	[33]
CuFe_2_O_4_	Co-precipitation	Traditional	[34]
CoFe_2_O_4_	Auto-combustion	Traditional	[35]
Cu: NiFe_2_O_4_ Thin films	Spray-pyrolysis	Traditional	[36]
CuFe_2_O_4_	Sol–gel self-combustion	Traditional	[37]
CuFe_2_O_4_ nanoparticles and CuFe_2_O_4_/PANI nanocomposites	Chemical precipitation with in-situ polymerization method	Traditional	[38]
CuFe_2_O_4_	Sol–gel self-combustion method with chelating agent modulation	Traditional	[39]
CuFe_2_O_4_	Auto-combustion	Traditional	[40]
CuFe_2_O_4_- Tragacanth gum	Sol–gel	Green chemistry	[41]
CuFe_2_O_4_- cow urine	Combustion	Green chemistry	[42]
CuFe_2_O_4_- *Morus alba* leaf extract	Sol–gel	Green chemistry	[43]
CuFe_2_O_4_/CFB (Cuttlefish bone) composite	Adsorption with catalytic wet air oxidation (CWAO)	Green chemistry	[44]
CuFe_2_O_4_- *Monsonia burkeana* extract	Hydrothermal	Green chemistry	[45]
Copper ferrite decorated reduced graphene oxide nanocomposite- *Azadirachta indica* leaf extract	Solution combustion	Green chemistry	[46]
CuFe_2_O_4_- Egg white	Auto-combustion	Green chemistry	[47]
CuFe_2_O_4_- *Chlorella* green microalgae extract	Hydrothermal	Green chemistry	[48]
Reduced graphene oxide decorated CuFe_2_O_4_- Spinach leaf extract	Green solution combustion	Green chemistry	[49]
CuFe_2_O_4_- Mushroom *Pleurotus florida* extract	Sol–gel	Green chemistry	Present study

Recently, the transition-metal oxide nanoparticles (NPs) have garnered momentous consideration due to their extensive applications [11]. Among their noteworthy uses are disinfectants for wastewater, bactericidal agents for next-generation batteries, optoelectronic devices, and catalytic processes [12–14]. Because of their atomic and electronic structures, these applications are determined by their physical and chemical properties [11, 15]. Nanocrystalline ferrites have been explored because of their unique electrical and structural characteristics. The medical applications of ferrite nanoparticles include antibiotics, MRI (magnetic resonance imaging), and hyperthermia [16, 17]. Iron and other transition metals, such as MnFe_2_O_4_, CoFe_2_O_4_, Zn/NiFe_2_O_4_, NiFe_2_O_4_, and, ZnFe_2_O_4_ among others, are found in spinel ferrites, which are ferromagnetic compounds [18]. In addition to their magnetic properties, they exhibit spin-canting effects, spin-glass-like behavior, and increased resistance to corrosion and heat [19]. The opto-electrical and magnetic characteristics of these NPs are remarkable. CuFe_2_O_4_ NPs are widely used as photocatalytic and magnetic materials [20]. Ferrites can enhance their characteristics by being combined with other metal ions [21]. Due to its numerous applications, Zn-NiFe_2_O_4_ and NiFe_2_O_4_ NPs have drawn attention [22]. Particularly in therapeutic applications, iron oxide nanoparticles (NPs)—especially ferrites—have recently garnered immense interest. In earlier investigations, CoNPs modified with Mn, Ni, Zn, and Cu have demonstrated enhanced antibacterial activity [23, 24]. Conjugating spinel ferrite NPs with other metal ions resulted in increased activity. Many scientific investigations have documented on the antibacterial characteristics of chromium-anchored CuFe_2_O_4_ NPs [25]. Due to their vast surface area, ferrite nanoparticles are good candidates for adsorption. By covering them with surfactants and enhancing their physicochemical stability for functionalized surface, NPs and their composites prevent aggregation [26]. In addition to its medical uses, their application in the remediation of organic and heavy metal contaminants has also been investigated [27].

Many medical improvements have been made possible by using fungicides and antibiotics to tackle infectious diseases. On the downside, these methods have reported notable increase in the number of organisms resistant to many drugs in the past few years. The effectiveness of current antibiotics is in jeopardy due to the increasing use of commercialized antimicrobial medicines in managing infectious illnesses and the genetic potential of bacteria to propagate and convey resistance to antimicrobial therapies [28]. The present-day antibiotics are primarily biological and inhibit a number of vital biochemical processes, such as the replication of DNA, the expression of genes, and the synthesis of biological membranes. But as troubling global AMR rates increased, researchers intensified their work and started examining and researching microbial biological systems as possible targets for future treatments. As part of this strategy, biologically active substances that come from natural sources need to be looked into. Antibacterial chemical discoveries can be made from various natural sources, including flora, animal species, prokaryotic cells, aquatic organisms, and fungi. It has been acknowledged that mushrooms are an effective ¨green¨ source of several antibacterial chemicals that can fight the aforementioned dangerous illnesses. In recent decades, they have become one of the most exciting prospects in the search for natural antibacterial medications. The mycelium and fruiting bodies both have antibacterial and antioxidant qualities. Many mushrooms have antibacterial properties, making them safe and effective medicines for serious illnesses. Many mushrooms, such as *Agaricus* sp., *Lentinus* sp., and *Pleurotus* sp., have exhibited potent antibacterial activity against microbial infections [28].

The most commonly grown and eaten lignocellulosic fungus is *Pleurotus*, or oyster mushroom. The group, which comprises about 40 species, is categorized as belonging to the class Basidiomycetes and the family Agaricaceae in the kingdom Mycota. Bioactive macromolecules are highly concentrated in oyster mushrooms [28, 29]. The concept of"food as medicine"underlies the use of mushrooms as a medicinal tool. The local populations have long utilized them—notably in South-East Asia—as food and medicine. Because of their nutritious qualities and frequent use as therapeutic agents, mushrooms are still utilized in the medical industry. As a result, scientists have examined mushrooms, and the isolation and clarification of the structure of bioactive chemicals serve as the primary focus of their research. As mushrooms have so many distinct medical uses, there has been an increase in the extraction of bioactive components from various mushroom species. Many biological potentialities, including antimicrobial, anti-inflammatory, antitumor, antioxidant, and anticancer properties, are provided by mushrooms, especially by their mycelium-containing medicinal compounds. Around the world, mushrooms are either naturally occurring or are farmed and frequently considered natural foods [30]. One of the most underappreciated foods of nutrient-rich and therapeutic value is mushrooms. They are a great source of crude fiber, sugars, proteins, minerals, vitamins, and oil fat. All of the major lipid groups—phospholipids, esters, sterols, mono-to triglycerides, and free fatty acids—are found in mushrooms. These bioactive fats derived from mushrooms have demonstrated a variety of biological effects. Mushrooms are a natural source of nutrients, antioxidants, and antibiotics. Among the antioxidants found in mushrooms are phenolic compounds (caffeic acid, tannic acid, catechin, benzoic acid, gallic acid and resveratrol), carotenoids, ascorbic acid, and tocopherols. On the other hand, antifungal, antiviral, and antibacterial activities of mushroom sesquiterpenes, glycolipids, and polysaccharides have been reported [28, 29]. The recent researches have focused on the significant pharmacological qualities of mushroom bioconstituents as potential anticancer therapeutic options. *Pleurotus* species can block growth, proliferation, angiogenesis, and metastasis of cancer cell line with extraordinary potency without harming healthy cells or suppressing the immune system. In summary, from the beginning of agriculture to the development of novel medications and non-conventional foods, efforts have been made to harness the natural advantages of fungi. In recent decades, the discovery of new chemicals originating from fungi that have the potential to be used as spectral band medications has gained significant attention [28]. Hence, keeping in view the aforementioned pros, the present work aims to synthesize CuFe_2_O_4_ NPs by sustainable process using the mushroom *Pleurotus florida*. The spectral analysis, optical studies, chemical composition analysis, and surface morphological aspects were used to characterize the biosynthesized nanoparticles. The bio-essential activities of antibacterial, anti-diabetic, antioxidant, anti-inflammatory and dye degradation were assayed by various experiments. Two different types of dye were used: The bright red dye, rhodamine B, is a fluorescent xanthene dye that is used in many industrial and biological applications. Methylene blue is a cationic dye that gives cells a blue stain as negatively charged particles including DNA, RNA, and polyphosphates are drawn to the positively charged dye. To my knowledge, the present work serves as first hand report of employing mushroom extract in the “green and clean” synthesis of CuFe_2_O_4_ nanoparticles.

## Materials and methods

### Collection and processing of plant material

Edible and fresh mushroom *Pleurotus florida* was procured from KVK (Krishi Vigyan Kendra), Thelliyoor and was maintained under ambient conditions. Copper (II) nitrate trihydrate [Cu(NO_3_)_2_.3H_2_O] and Iron (III) nitrate nonahydrate [Fe(NO_3_)_3_.9H_2_O] (Analytical grade) were purchased from Sigma-Aldrich. The aqueous extract of *Pleurotus florida* (PFE), an edible mushroom, was made using a slightly modified version of Priyadarshni and Mahalingam [50]. Following, 20 g of fresh mushroom samples were cleaned with sterile distilled water, allowed to air dry, and then roughly chopped with a sterile knife; they were combined with 100 ml of double distilled water and vigorously agitated for almost half an hour. After heating the combination at 50 °C for 20 min, the extract (PFE) was filtered using Whatman No. 1 filter paper. In the biosynthesis of CuFe_2_O_4_NPs, the PFE was employed as a stabilizer and reducer.

### Synthesis of PFE-CuFe_2_O_4_ NPs

**Fig. 1:**
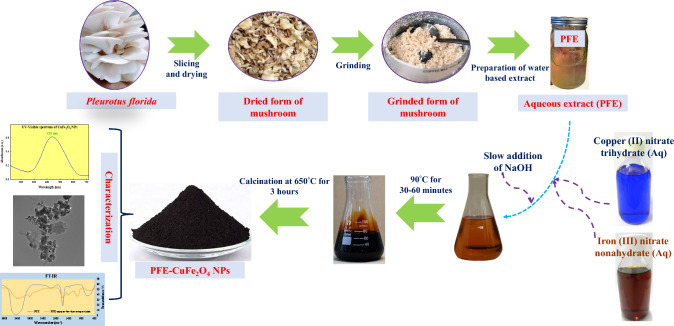
Diagrammatic representation of synthesis of PFE-CuFe_2_O_4_ NPs

**Fig. 2:**
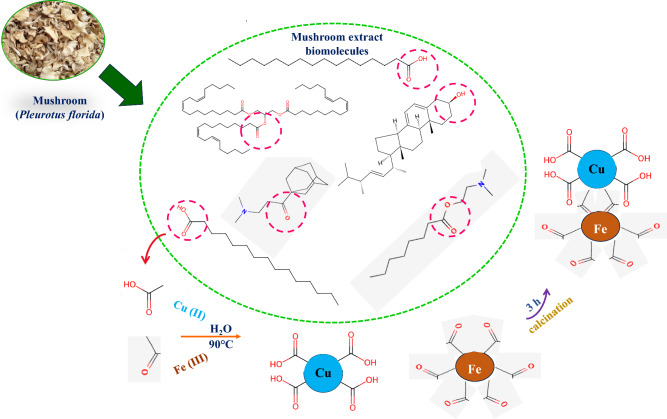
Mechanism of synthesis of PFE-CuFe_2_O_4_ NPs

Separate solutions of Fe(NO_3_)_3_.9H_2_O (0.1 M) and Cu(NO_3_)_2_.3H_2_O (0.05 M) were prepared. A mechanical stirrer was used for two hours to mix 20 ml of the combination comprising 10 ml each of ferric and copper nitrate with 30 ml PFE. Slow additions of ammonium hydroxide solution were made to stabilize pH 8.0. The mixing operation was carried out using magnetic stirrer to ensure the fluid was homogenized. The temperature was raised to 90 °C and held there—a gel created with constant shaking. The gel eventually turned into a dry gel (Xerogel), which was then thermally calcined for three hours at 650 °C to produce CuFe_2_O_4_ NPs [41, 47, 51]. The diagrammatic representation of synthesis and mechanism are pictorially illustrated in Figs. 1, 2, 18.

### Morphological and physico-chemical characterization

The vibrational, structural, and morphological characteristics of CuFe_2_O_4_ NPs were investigated using characterization techniques of zeta potential, FT-IR (Fourier transform infrared spectroscopy), UV–visible spectroscopy, XRD, and electron microscopy (TEM, SEM), GC–MS. The operational specifications were followed as mentioned in the studies of Cherian et al. [52].

### Biological activities of CuFe_2_O_4_ NPs

The biological activities of CuFe_2_O_4_ NPs: anti-diabetic (inhibition of enzymes α-amylase and α‑glucosidase) [53, 54], antioxidant (DPPH, TRP, TAC, ABTS) [55–57], anti-inflammation (COX-1, COX-2, 15-LOX, sPLA2) [58], anti-bacterial, ROS production, ETSA inhibition, and anti-biofilm [59–64], molecular docking [65–70] was ascertained. The detailed information of assays is mentioned in Supplementary file S1.

### CuFe_2_O_4_ NPs assisted dye degradation studies

The degradation of dyes Methylene blue (MB) and Rhodamine B (RhB) was examined using CuFe_2_O_4_ NPs as nanocatalysts. As monitor wavelengths, the dyes MB and RhB exhibit absorbance maxima at 668 and 541 nm, respectively. The dye solutions were agitated for 20 min in the dark after adding the proper quantity of photocatalyst (CuFe_2_O_4_ NPs) to achieve adsorption/desorption equilibrium. After shaking the mixture manually, the absorption spectra were recorded at room temperature. The absorbance change over time was used to quantify the catalytic degradation. The % degradation was computed by the following equation:

$${\text{D }} = \, \left( {{\text{C}}_{{\text{o}}} - {\text{C}}_{{\text{t}}} } \right)/{\text{C}}_{{\text{o}}} \times {1}00$$

where C_o_ and C_t_ = dye concentrations at time 0 min and t min, respectively.

### Statistical analysis

The experimental values were reported as mean ± standard deviation for each experiment run in triplicates. The *p* value (*p* ≤ 0.05) was considered as level of significance.

## Results and discussion

Green synthesis and biogenic reduction are "Bottom Up" techniques that replace reducing agents with green extracts that have inherent stabilizing, finishing, and capping properties. The precise synthetic pathway involved in the creation of biomimetic nanoparticles has not yet been fully determined. According to earlier researches, biological compounds and other phytochemicals present in the biological extracts effectively functions as reducing agents when they combined with metal precursors to generate nanoparticles [71–73]. The production of nanoparticles by biosynthesis is anticipated to involve the contributions of several biomolecules rather than just one. Therefore, the reaction pathway may differ for various biological extracts or for the same extract depending on the extraction solvent used. Other variables that can also be significant include pH and the location of the biological samples [73]. The mechanism of biological extract-based nanoparticle production consists of three primary phases: (a) the activation phase, when metal atoms start to nucleate and metallic ions are reduced; (b) Ostwald ripening- the growth phase where the metal atoms group together to create larger particles (nanoparticles are generated immediately by heterogeneous nucleation, growth and development, subsequently leading to ion reduction); this increases the thermodynamic stability of the nanoparticles, and (c) phase of termination which establishes the definitive morphology of nanoparticles.

Ferrites are constructed based on the reaction between iron oxides and another oxide of a divalent element in the solid–solid contact. The ferrous ion is one of the iron ions that should be in the reaction medium since it works in concert with other iron ions to drive these reactions and produce the associated ferrites [74]. This objective can be accomplished by employing natural extracts which provides milieu of reducing and stabilizing biomolecules. This natural milieu will convert some ferric ions into ferrous ions, which work in concert with one another [74, 75]. Figures 2 and 18 depicts a classified framework for the production of CuFe_2_O_4_ NPs using mushroom extract. The following describes the precise mechanism of the solid–solid contact between the stoichiometric oxides of Cu and Fe resulting in the formation of CuFe_2_O_4_ NPs [74]. (i) Two processes take place at the ferric oxide contact. Fe_2_O_3_ and the divalent metal ions (M^2+^ = Cu^2+^) interact in the first operation to produce Fe^2+^ ions and the associated ferrite-based thin layer, which envelops Fe_2_O_3_ and prevents the solid–solid contact between the interacting oxides from reaction progression. In the second operation, some Fe_2_O_3_ and carbon react in the presence of mushroom biomolecules, producing more Fe^2+^ ions. (ii) At its interface, the resultant Fe^2+^ ions can undergo another reaction with the copper compound to generate an excess of the ferrites under investigation along with Cu^2+^ ions, which then return for the completion of solid–solid interaction.

**Fig. 3:**
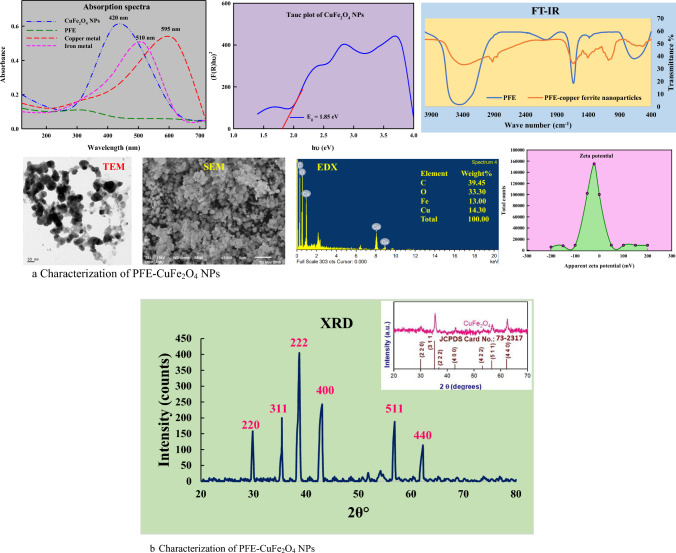
**a** Characterization of PFE-CuFe_2_O_4_ NPs. **b** Characterization of PFE-CuFe_2_O_4_ NPs

One simple method to confirm the emergence of metal nanoparticles, assuming the metal has surface plasmon resonance (SPR), is to examine the UV–visible spectrum. The size, form, dispersion of the particles in water-based solutions primarily determine the shape and location of the SPR band of nanoparticles. The UV–Vis absorption spectrum of CuFe_2_O_4_ NPs is shown in Fig. 2. In PFE-CuFe_2_O_4_ NPs, the highest absorbance peak was measured at 420 nm. Jabeen et al. [27] reported the absorbance spectra of *Cissus rotundifolia* assisted CuFe_2_O_4_ NPs at 421 nm while the optical absorption spectra of CuFe_2_O_4_ NPs revealed an absorption peak at 409 nm in prior research of Meidanchi and Ansari [76]. In Fig. 3a, the predicted band-gap value for CuFe_2_O_4_ NPs was determined to be 1.85 eV, which was nearly identical to the previously published literature of Park et al. [77]. For calculating the band gap energy, Tauc's Eq. was used:

$$\left(\propto hv\right)n=A(hv-Eg)$$

where hν is the incident photon energy, A is the absorbance, Eg is the band gap energy, and n = ½ and 2 for direct and indirect band gap, respectively.

FTIR analysis was carried out in the present investigation of PFE and biosynthesized CuFe_2_O_4_ NPs. The interaction of the PFE’s functional groups with the surface of environmentally friendly CuFe_2_O_4_ NPs is demonstrated in Fig. 3a. The biologically active compounds that work as a capping and stabilizing agent during the formation of CuFe_2_O_4_ NPs were verified when functional groups in an FTIR spectrum of PFE were identified [51]. The spectra showed two significant absorption bands: 402 cm^−1^ (octahedral stretch of Cu–O) and 591 cm^−1^ (intrinsic stretch at the tetrahedral position of Fe–O). The band observed at 3340 cm^−1^ was ascribed to the stretch vibrations in OH group. Similar to spinel CuFe_2_O_4_ nanocrystals previously described by Zakiyah et al. [78], the distinctive bands were roughly in the same range, with the bands resulting from Cu–O and Fe–O stretch observed at 422 and 586 cm^−1^, respectively. The C–O–C stretch was observed at 1339 and 1115 cm^−1^, whereas the bending vibration of CH_2_ group was reported at 1467 cm^−1^. According to earlier reports, the peak observed at 2926 cm^−1^ was connected to alkyl group's C–H stretch [79].

Using electron microscopic examinations, the morphological characteristics of CuFe_2_O_4_ NPs were determined. The SEM micrograph (Fig. 3a) of CuFe_2_O_4_ NPs with a broadly spherical morphology composed of minuscule particles and nanocrystallites and a non-uniform NPs distribution [80]. The EDX result revealed the composition of the elements of CuFe_2_O_4_ NPs as carbon (C) (39.45%), oxygen (O) (33.3%), copper (Cu) (14.30%,), and iron (Fe) (13%), respectively. The outcomes were consistent with the earlier published researches of [27, 81, 82]. On other hand, the sample was prepared for TEM investigation by dispersing diluted droplets of CuFe_2_O_4_ NPs in ethanol on carbon-coated Cu grids and subsequently dried at room temperature. Figure 3a depicts the TEM image of CuFe_2_O_4_ NPs, which displays an even morphology and an approximately spherical form free of agglomeration [83]. The average particle size was found to be 22 ± 1.4 nm. As seen in Fig. 2, SEM and TEM investigations demonstrated the crystallinity of nanoparticles [84].

The value of zeta potential (ζ) commonly corresponds to the surface charge of nanoparticles. Elevated or decreased ζ values suggest varying degrees of electrostatic repulsion between the nanoparticles. The CuFe_2_O_4_ NPs aggregate because the magnetic attraction outweighs the electrostatic repulsion. Agglomeration causes zeta value to increase, which causes nanoparticles to be repelled electrostatically. The ZP, or negative potential, was determined to be − 28.9 ± 0.2 mV, meaning that CuFe_2_O_4_ NPs have an overall negative charge (Fig. 3a). The zeta potential of CuFe_2_O_4_ NPs demonstrated excellent electrostatic repulsion of particles, avoiding NP aggregation and colloidal integrity in aqueous solutions. The CuFe_2_O_4_ NPs have good colloidal stability at neutral pH, which makes them appropriate for medicinal applications [85].

The XRD analysis of CuFe_2_O_4_ NPs (Fig. 3b) reported 2θ peaks at = 29.86^◦^, 35.43^◦^, 38.74^◦^, 43.11^◦^, 56.90^◦^, and 62.28^◦^ ascribed to hkl planes (220), (311), (222), (400), (511) and (440); matched with JCPDS file no. 73–2317. The average particle crystallite size (D) was found to be 21 ± 0.93 nm for the maximal intensity peak in the (222) plane. The findings were found to be in alignment with the reports of Jabeen et al. [27] and Leichtweis et al. [86].

The biomedical field has focused a lot of attention on active compounds that have been extracted from naturally occurring biosystems such as algae, lichens, higher plants, and mushrooms in recent decades due to their no serious side-effects, low toxicities, and wide range of therapeutic properties [87]. A common and economically significant mushroom in temperate, subtropics, and tropics locations across the world, the *Pleurotus* mushroom is a member of the family Pleurotaceae [88]. Numerous vital nutrients, such as dietary fiber, protein, amino acids, vitamins, and minerals, are present in *Pleurotus* sp. [89]. With their antimicrobial, hypocholesterolic, hematological, anticancer, and immunomodulatory properties, *Pleurotus* sp. shows promise as therapeutic mushrooms [90]. The edible mushroom *P*. *florida* is gaining popularity as a nutritious, high-protein vegetable due to its exceptional flavor and taste. Because of its inexpensive cost and the ease of access to a variety of cultivation substrates, it is widely popular as cultivable species. In traditional medicine, using mushrooms to treat a variety of illnesses, including cancer, has been prescribed [87]. The GC–MS analysis classified the presence of 14 compounds in the ethanolic extract of *Pleurotus florida* (Table 2; Chromatogram mentioned in Supplementary file S1). The major compound of 9,12-Octadecanoic acid, methyl ester covered the peak area of 26.17% followed by Ergosterol (21.47%), Hexadecanoic acid (17.93%), Octadec-9-enoic acid (12.31%), 1(1-Adamantyl)−3-(Dimethylamino)−1-Propanone (5.24%).

**Table 2:** GC–MS analysis of PFE

S no	Compounds	Retention time (minutes)	Area%
C1	3-Hydroxy2-methylGamma Butyrolactone	8.023	0.93
C2	Diethyl1-methyl-3-hydroxy-5-phenylpyrrole-2,4-dicarboxylate	8.142	2.22
C3	1,2,3-Benzenetriol	11.365	1.48
C4	Pentadecanoic acid	17.119	2.42
C5	Hexadecanoic acid	18.984	17.93
C6	9,12-Octadecanoic acid, methyl ester	23.022	26.17
C7	Octadec-9-Enoic acid	23.104	12.31
C8	Cysteamine S-Sulfate	23.558	2.15
C9	1(1-Adamantyl)−3-(Dimethylamino)−1-Propanone	29.274	5.24
C10	Octanoicacid,2-dimethylaminoethyl ester	29.342	1.00
C11	1,2 Benzene dicarboxylic acid	30.918	2.80
C12	Trilinolein	33.150	2.71
C13	Ergosterol	42.176	21.47
C14	9(11)-Dehydroergosteryl benzoate	37.416	1.17

**Fig. 4:**
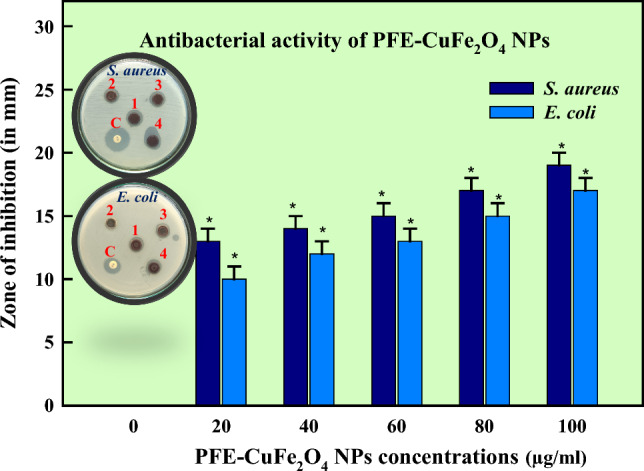
Antibacterial activity of PFE-CuFe_2_O_4_ NPs (C = control; 1 = 20 µg/ml; 2 = 60 µg/ml, 3 = 80 µg/ml, 4 = 100 µg/ml)

**Fig. 5:**
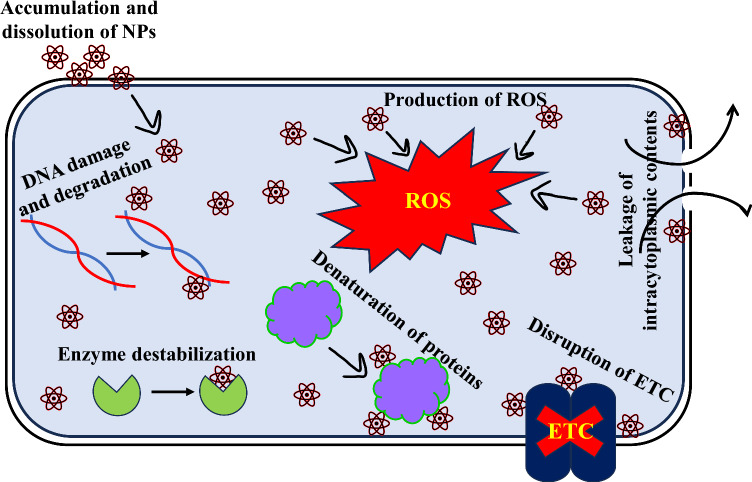
Antibacterial mechanism of PFE-CuFe_2_O_4_ NPs

**Fig. 6:**
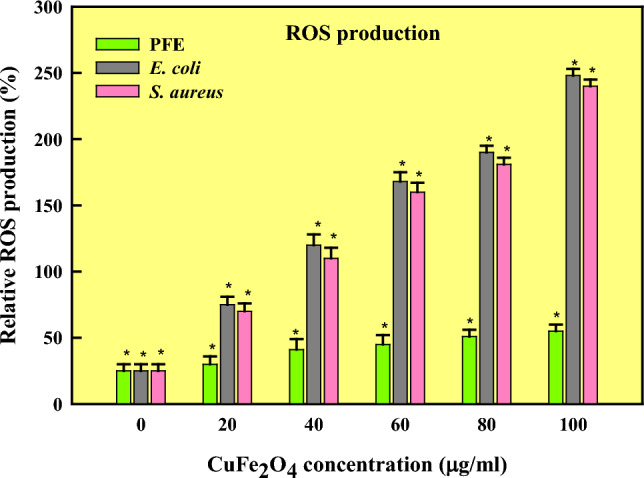
ROS production by PFE-CuFe_2_O_4_ NPs

**Fig. 7:**
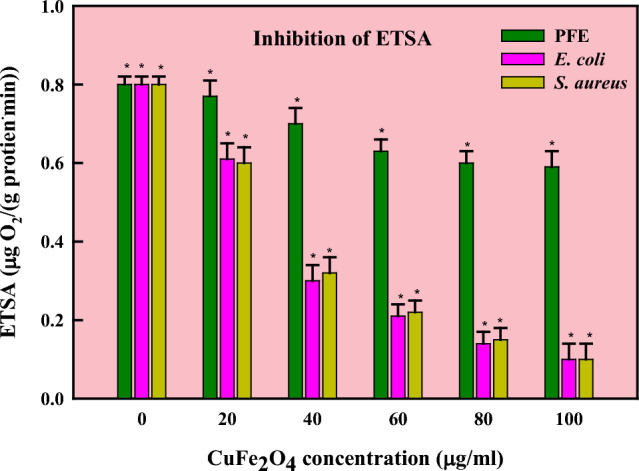
ETSA inhibition by PFE-CuFe_2_O_4_ NPs

**Fig. 8:**
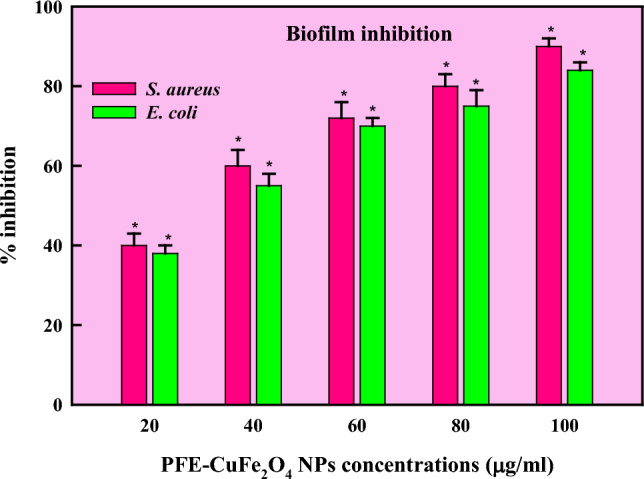
Antibiofilm activity of PFE-CuFe_2_O_4_ NPs

Natural sources are the primary source of antibiotics, obstructing vital biological processes such as cell membrane formation, translation, and reproduction. Scientists have, therefore, started to investigate and evaluate microbes'metabolic processes as possible therapeutic targets in response to the increasing antibiotic resistance observed globally [91]. As part of this strategy, therapeutic molecules made from biological materials must be researched. Prokaryotes and eukaryotes have a variety of natural sources from which antimicrobial chemicals can be found [92]. Mushroom extract is a well-known natural reservoir of several antimicrobial substances which are used in the treatment and management of serious infections. Previous research revealed that Basidiomycota and Ascomycota had strong antibacterial qualities against bacterial infections. Chemicals derived from various microorganisms, most notably edible mushrooms, have many pharmacological and biological properties, such as antibacterial, nutritional components, immunomodulation, free radical scavenging, antihyperglycemic, and anti-carcinogenic properties [28]. The antibacterial potential of CuFe_2_O_4_ NPs was ascertained against the pathogenic strains of *S. aureus* and *E. coli* (Fig. 4) and compared with antibiotic Gentamycin [93]. The activity as implied in form of inhibitory zone reported as: *E. coli* (17 ± 1.0 mm) and *S. aureus* (19 ± 1.5 mm) at 100 µg/ml. A comparative account of antibacterial and antioxidant activities of copper ferrite nanoparticles has been mentioned in Table 3. In Fig. 5, an accumulation of nanoparticles in bacterial samples might lead to denaturation of the cell membrane, compromising the antibacterial activity [94]. Gram-positive bacterial cells are more vulnerable to the toxicity of ferrite nanoparticles than Gram-negative ones as they have a denser peptidoglycan coating [95]. An organism's ability to transport, breathe, metabolize energy, lyse cells, and eventually dies is compromised when it produces reactive oxygen species (ROS) [96]. The prokaryotic cells become more cytotoxic when ROS are produced as the latter cause oxidative stress, which destroys proteins, lipids, membranes, and nucleic acids. The ROS production was found to be in appreciable amounts in both the cells (Fig. 6) yet a slightly more pronounced effect was observed in *E. coli* cells than *S*. *aureus* cells. The findings imply that the interaction of CuFe_2_O_4_NPs with various bacterial cells is what causes the differential generation of ROS. Through toxicity and oxidative stress, the contact alters the cellular structure; affecting protein synthesis and leading to cell death. The CuFe_2_O_4_/Ti_3_C_2_ nanocomposite was shown in a study of Reddy [97] to have better antibacterial activity against pathogenic strains than CuFe_2_O_4_ and Ti_3_C_2_ NPs alone. Moreover, CoFe_2_O_4_/SiO_2_'s antibacterial qualities demonstrated the most potent inhibitory effect against *E*. *coli* and *S*. *aureus* [98]. In a different investigation, ferrite NPs combined with nickel and copper exhibited superior antibacterial efficacy to NiFe_2_O_4_ against pathogenic strains [83]. The Electron transport system activity (ETSA) of bacterial cells dramatically dropped when exposed to CuFe_2_O_4_ NPs stress (Fig. 7). When the bacterial cells were exposed to CuFe_2_O_4_ NPs, its ETSA dropped to 48.8% and 30.0% of the control. While the NPs can harm bacterial cell membranes and membrane proteins, the quinones in the inner membrane of bacteria serve as electron donors for biochemical reduction [99, 100]. As a result, the bioenergetic reactions exhibited limited metabolic activity as the electron transfer was inhibited. In addition, the management of bacterial infections is facing a serious dilemma because of the bacterial capacity to create biofilms and develop structures resistant to traditional medications. Therefore, creating efficient antibacterial substitutes to fight these bacteria is crucial. This study presents a novel approach to prevent biofilm forming bacterial strains by employing mushroom-mediated manufactured CuFe_2_O_4_ NPs. Compared to the growth control, the biosynthesized CuFe_2_O_4_ NPs effectively prevented the growth of biofilm formers at all concentrations. The % inhibition of CuFe_2_O_4_ NPs against biofilm forming strains of *S*. *aureus* and *E*. *coli* was reported to be 90 ± 1.2% and 84 ± 1.4%, respectively (Fig. 8). Concurring with our results, the quasi-spherical-shaped silver nanoparticles (80–120 nm diameters) synthesized by *Solibacillus isronensis* sp. reported noteworthy biofilm inhibitory action against *E. coli* at 1.56 μg/ml AgNPs [101]. Chandrasekharan et al. [102] reported 46% biofilm inhibition by biofabricated hydrogel- *Gmelina arborea* mediated spherical AgNPs. Singh et al. [103] reported pronounced anti-biofilm activity of *Rhodiola rosea* rhizome extract mediated AgNPs at a concentration > 6.25 μg/ml. Additionally, several mechanisms have been proposed highlighting nanoparticle mediated biofilm inhibitory strategies at sub-MIC levels which might be explained by their inhibition of gene expression in biofilm formation [104]. The natural ability of mushrooms to produce anti-bacterial chemicals has led to their increasing significance as a potential alternative to chemically fabricated additives, whose benefits for healthiness and wellness have long been contested. Vigorous antibiotic activity has been shown for a variety of mushrooms against pathogenic bacteria that are resistant to multiple drugs, including *Mycobacterium tuberculosis*. An innovative approach to managing tuberculosis infection is using mushroom fungal extracts, which inhibit tuberculosis bacteria instead of prescribed cytotoxic medications [105].

**Table 3:** Comparative study of antibacterial and antioxidant properties of CuFe_2_O_4_ NPs

Composition of nanoparticles	Biological properties	Application	References
Cu- *Hibiscus sabdariffa* petal extract @Fe_3_O_4_	Antioxidant	DPPH activity (90% at 1000 µg/ml)	[112]
CuFe_2_O_4_- *Cissus rotundifolia* leaf extract	Antibacterial	*B*. *subtilis* and *B*. *pumilis* (inhibition zone of 12 ± 0.2, 11 ± 0.4 at 75 mg/ml, respectively); *E*. *coli*, *P*. *aeruginosa* and *S*. *abony* (inhibition zone of 14 ± 0.4, 18 ± 0.3, 12 ± 0.2 at 75 mg/ml, respectively)	[27]
Cu@Fe_3_O_4_/SiO_2_@MWCNTs	Antioxidant	DPPH activity	[113]
Cu/Fe_3_O_4_- *Helleborus niger* extract	Antioxidant	DPPH activity	[114]
Cu/Fe_3_O_4_- Green tea extract	Antioxidant	DPPH activity (90.25% at 1000 µg/ml)	[108]
Copper ferrite decorated reduced graphene oxide nanocomposite- *Azadirachta indica* leaf extract	Antibacterial	*Bacillus cereus* (inhibition zone 7–15 mm at 2.5–10 mg/ml) and *Pseudomonas aeruginosa* (inhibition zone up to 80 mm at 10 mg/ml)	[46]
CuFe_2_O_4_- *Chlorella* green microalgae extract	Antibacterial	Inhibition of *Staphylococcus aureus* and *Escherichia coli*	[48]

**Fig. 9:**
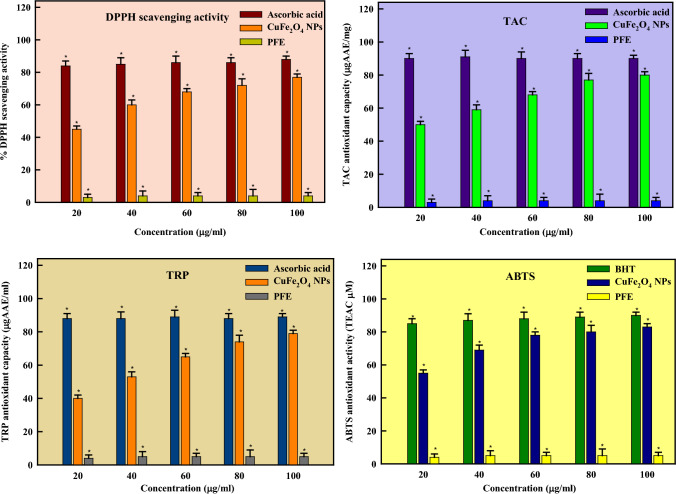
Antioxidant activity of PFE-CuFe_2_O_4_ NPs

An improper balance between excessive nitrogen synthesis and reactive oxygen species causes cellular oxidative stress. Antioxidants are essential for ceasing chain reactions that might injure vital biological components and interfere with cellular functions, hence mitigating the negative effects of reactive species. Antioxidants are compounds that undergo additional oxidation to form stable, non-reactive intermediates. They fall into two primary classes: (i) preventive antioxidants that prevent the formation of new radical chains and (ii) chain-breaking or radical-trapping antioxidants that trap chain-carrying radicals and break the oxidation chain [106]. Antioxidant substances derived from biological extracts are essential for reducing the incidence of infectious illnesses [107]. Nanoparticles are useful for a variety of medical applications because they aggressively seek out and destroy free radicals. This effectiveness is especially apparent when treating illnesses brought on by ROS that throw off the normal redox balance [106]. According to previous reports, copper-based nanocomposites have excellent antioxidant qualities, as demonstrated by various radical scavenging experiments [108]. This has strengthened the application of copper-based nanocomposites as useful antioxidants in the treatment of illness [108]. Using the radical scavenging DPPH, TAC, TRP, ABTS techniques, the antioxidant qualities of PFE and CuFe_2_O_4_ NPs were assessed. Stable chemical DPPH was employed to evaluate the antioxidant properties; it reduces as it accepts hydrogen or electrons [107]. A lower IC_50_ value indicates a stronger scavenging ability of the produced NPs or extract, whereas a larger IC_50_ value indicates a lower scavenging activity of the compounds [107]. The effects of different CuFe_2_O_4_ NPs and PFE concentrations in addition to regular ascorbic acid were examined (Fig. 9). The results indicated that the PFE exhibited low scavenging properties at varying doses. In contrast to the PFE, the manufactured CuFe_2_O_4_ NPs demonstrated more pronounced scavenging activity. In comparison to the stable DPPH molecule, it demonstrates the dose-dependent (20–100 µg/ml) reaction of CuFe_2_O_4_ NPs, PFE, ascorbic acid. The CuFe_2_O_4_ NPs demonstrated scavenging activity ranging from DPPH (77 ± 2.1%), TAC (80 ± 1.5%), TRP (79 ± 2.5%), ABTS (83 ± 1.2%). The manufactured NPs demonstrated pronounced scavenging activity, which may be elucidated by the presence of capped flavonoids, tannins, and phenolic compounds in green-produced NPs [109]. As phenolic compounds have more outstanding redox capabilities for trapping and eliminating free radicals, they exhibit significant antioxidant activity [110]. The neutralization of free-radical nature of DPPH through electron transfer is the primary cause of the metal-based nanoparticles mediated antioxidant properties [111]. Moreover, the high surface to volume ratio of metal-based nanoparticles may be responsible for their antioxidant properties [111]. Generally speaking, oxidative stress limits biomaterial biocompatibility and causes major issues including inflammation and chronic illnesses. Therefore, the concentration-based antioxidant activity that our investigation documented is encouraging and offers a starting point for investigating copper ferrite nanoparticles as a potential novel antioxidant nanocomposite.

**Fig. 10:**
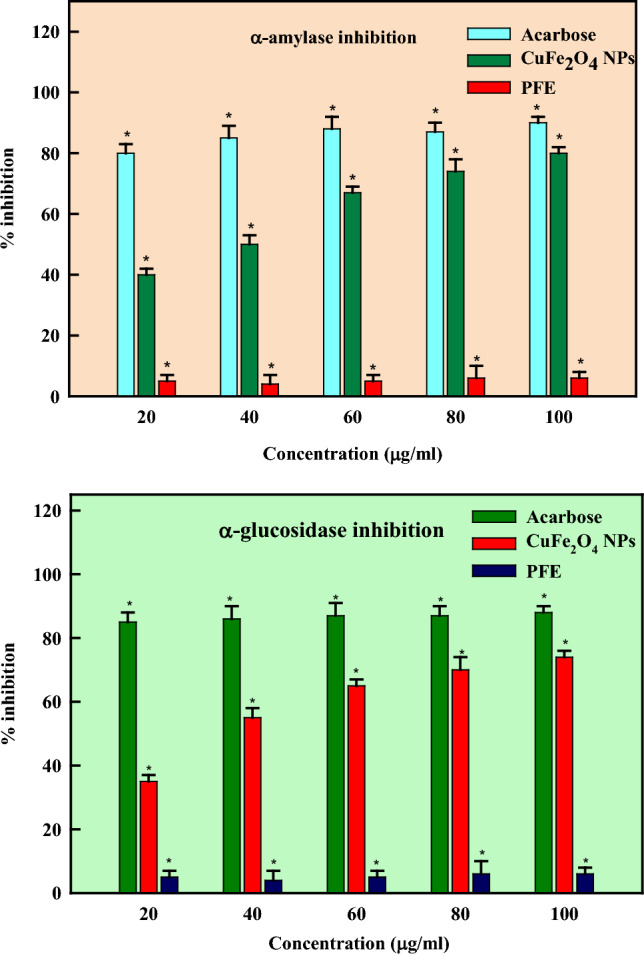
Antidiabetic activity of PFE-CuFe_2_O_4_ NPs

A complex set of conditions termed as DM (diabetes mellitus) type II affects the process of carbohydrate metabolism either by diminished tissue sensitivity to insulin or by insufficient insulin production [115–117]. DM type II ranks sixth among the world's top causes of death. Several countries use numerous plants as traditional medicines to treat type II diabetes [118]. This study studied the ability of CuFe_2_O_4_ NPs with established anti-diabetic action to inhibit the activities of enzymes α-amylase and α-glucosidase. The CuFe_2_O_4_ NPs were examined individually and in combination at varying doses (20–100 μg/ml) (Fig. 10). The CuFe_2_O_4_ NPs exhibited the highest level of inhibition at 100 μg/ml, with 80 ± 2.9% and 74 ± 3.1%, compared to the acarbose (88.20 ± 3.0% and 90.2 ± 1.5%) for α-amylase and α-glucosidase, respectively. In agreement with our results, Barabadi et al. [104] synthesized aqueous seed extract of *Pimpinella anisum*-AgNPs and exhibited a pronounced concentration-dependent inhibition of α-amylase to the tune of 44.90 ± 3.87 and 71.43 ± 4.92% at 0.25 and 1 mg/ml concentrations at 1 mg/ml concentration. Perumalsamy and Krishnadhas [118] reported α-amylase inhibition of 52.48% at 1 mg/ml polygonal silver nanoparticles synthesized by the seed extract of *M. fragrans*. Sarfraz et al. [119] reported 60–80.52% α-amylase inhibition by *Mentha longifolia* and *Zingiber officinale* leaf extract mediated silver nanoparticles at 1 mg/ml concentration. Andleeb et al. [120] synthesized spherical AgNPs mediated by the clove extract of *Allium sativum* and reported α-amylase inhibition to the tune of 44.24 ± 0.64% at 0.5 mg/ml concentration. Similarly, the leaf extract of *Solanum khasianum* biofunctionalized spherical silver nanoparticles exhibited α-amylase inhibition (79.56%) at 1 mg/ml concentration [121]. The leaf extract of *Muntingia calabura* biosynthesized spherical silver nanoparticles reported α-amylase inhibition of 78.16% at 1 mg/ml concentration [122]. α-amylase is the enzyme that converts complex carbohydrates like glucose into simpler sugars. The inhibition of α-amylase activity can cause an extended-release of glucose into the blood circulation after a meal by slowing down the rate at which carbohydrates convert to glucose [123]. The NPs can attach themselves to enzymes and hinder their regular activity, which lowers the pace at which carbohydrates are broken down and glucose is released. Another enzyme that aids in the digestion of carbohydrates is α-glucosidase, which converts complex carbohydrates into simpler sugars that the body can absorb and use as fuel [124, 125]. The intestinal rate of glucose absorption can be decreased by inhibiting the activity of enzyme α-glucosidase, which eventually slowdowns the transformation of complex carbohydrates into glucose. By reducing the conversion of complex sugars into glucose and interfering with α-glucosidase's enzymatic activity, the nanoparticles can interact with the enzyme. The green synthesized CuFe_2_O_4_ NPs helped in controlling the blood sugar levels by slowing down the pace at which carbohydrates are broken down, and glucose is absorbed by blocking the actions of the enzymes α-amylase and α-glucosidase [126]. These qualities make them attractive candidates for developing all-natural, environmentally benign anti-diabetic medications.

**Fig. 11:**
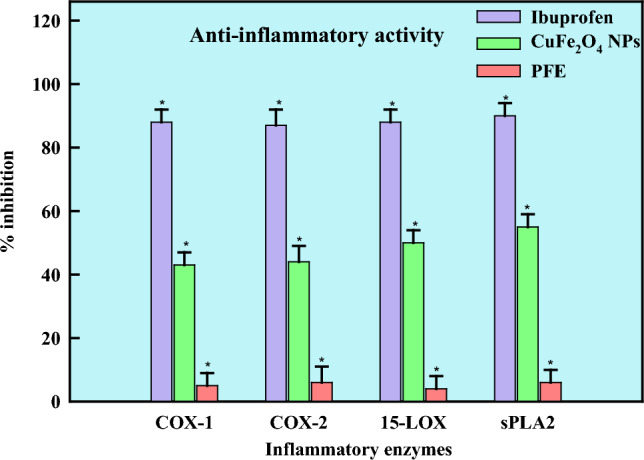
Anti-inflammatory activity of PFE-CuFe_2_O_4_ NPs

A pathological state known as inflammation is responsible for reestablishing tissue microenvironment and cellular homeostasis following injury and infection [127]. Chronic inflammation is the cause of conditions like pancreatitis, asthma, inflammatory bowel disease, and rheumatoid arthritis [128]. The use of economically accessible NSAIDs (non-steroidal anti-inflammatory medicines) to treat chronic inflammatory illnesses is limited due to their numerous side effects, which include bleeding, ulcers in the stomach, disruption of the gastric mucosal layer, and cardiac strokes [129]. Most NSAIDs exhibit low water-based solubility limiting the absorption of medicines via cellular membranes by affecting the rate at which medicines dissolve in blood plasma [130]. Because of their delayed absorption in cases of acute inflammation, NSAIDs take longer to start having anti-inflammatory, analgesic, and antipyretic actions. Numerous prior studies indicate that the direct interface between the mucosal epithelium and acidic NSAIDs may cause gastrointestinal adverse effects. Therefore, there is a critical need to create new nanoformulations that have improved anti-inflammatory qualities while minimizing adverse effects. The in-vitro pathways for COX-1, COX-2, 15-LOX, and sPLA2 were used to identify anti-inflammation effects of CuFe_2_O_4_ NPs synthesized by *Pleurotus florida*. All pathways were shown to exhibit effective inhibitory effects. The maximal inhibition was reported against sPLA2 (55 ± 2.6%), followed by 15-LOX (50 ± 2%), COX-2 (44 ± 2.5%), and COX-1 (43 ± 2.5%), respectively (Fig. 11). Anti-inflammatory activity refers to a feature of medications that might lessen edema or inflammation. Mushrooms possess several beneficial constituents responsible for their anti-inflammatory properties—increased flow of blood to the wounded tissues resulting in increased temperature, edema, redness, discomfort, which is the tissue mediated response to the injury. The anti-inflammatory mechanism and the interaction between nanoparticles and cells are enhanced by metal nanoparticles [131]. It is a helpful reaction in the event of an unintentional wound injury and subsequent infection [132].

**Fig. 12:**
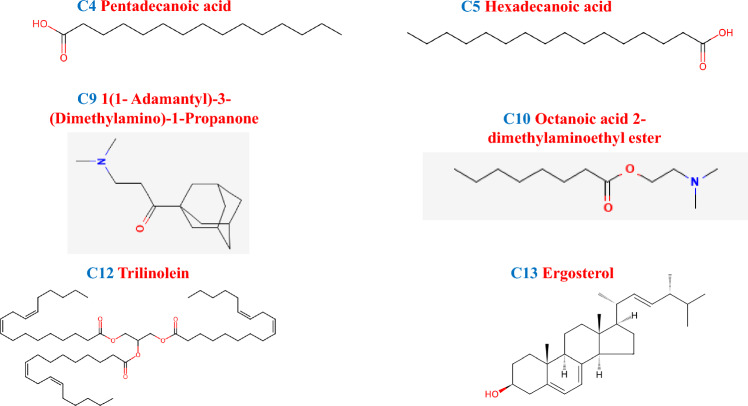
Compounds present in PLE exemplified by GC–MS

**Fig. 13:**
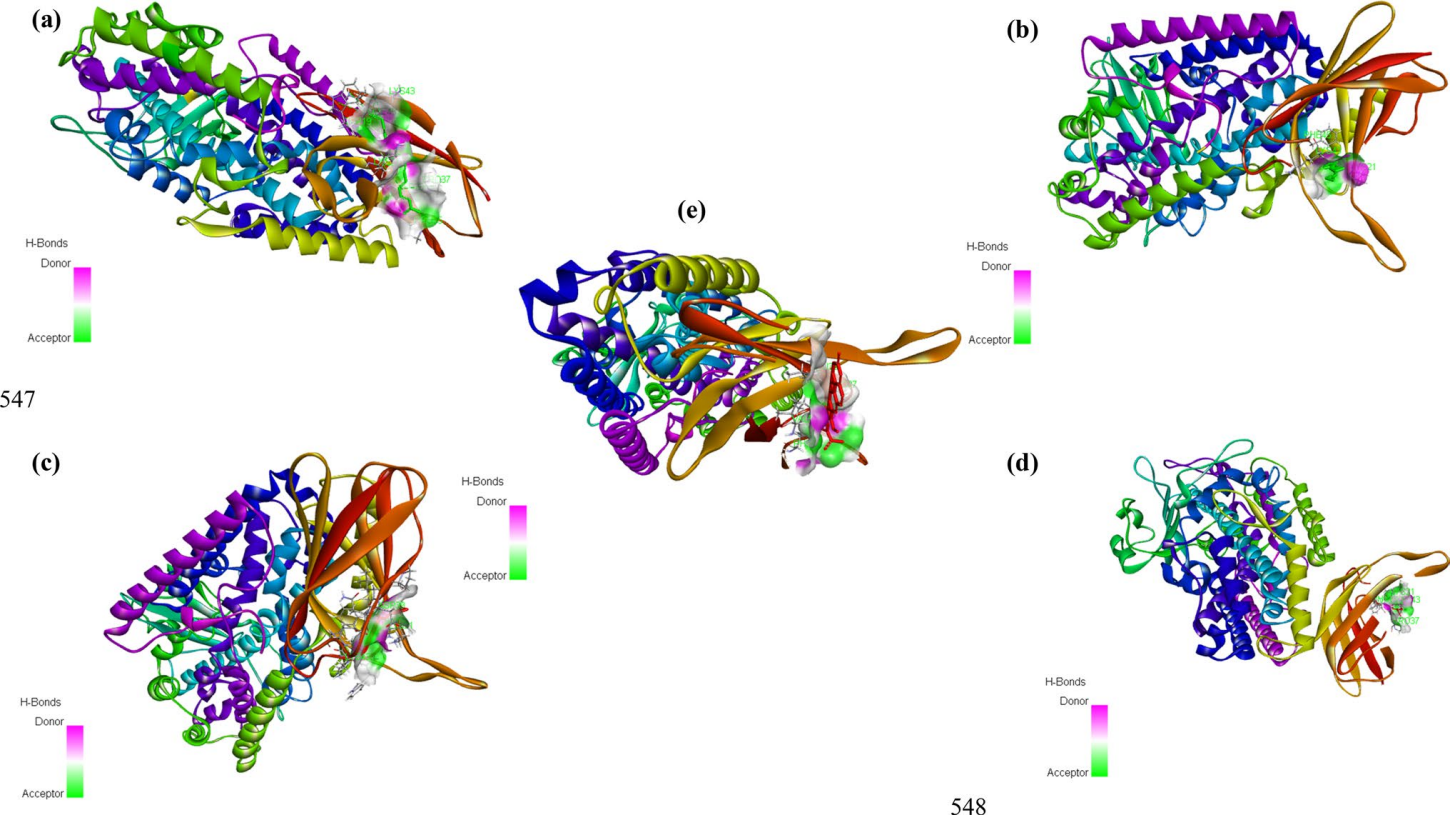
The docked pose and 2D image of **a** Pentadecanoic acid (C4); **b** 1(1- Adamantyl)−3-(Dimethylamino)−1- Propanone (C9); **c** Octanoic acid, 2-dimethylaminoethylester (C10); **d** Hexadecanoic acid (C15); **e** Ergosterol (C14) with the active site of 15-LOX

**Fig. 14:**
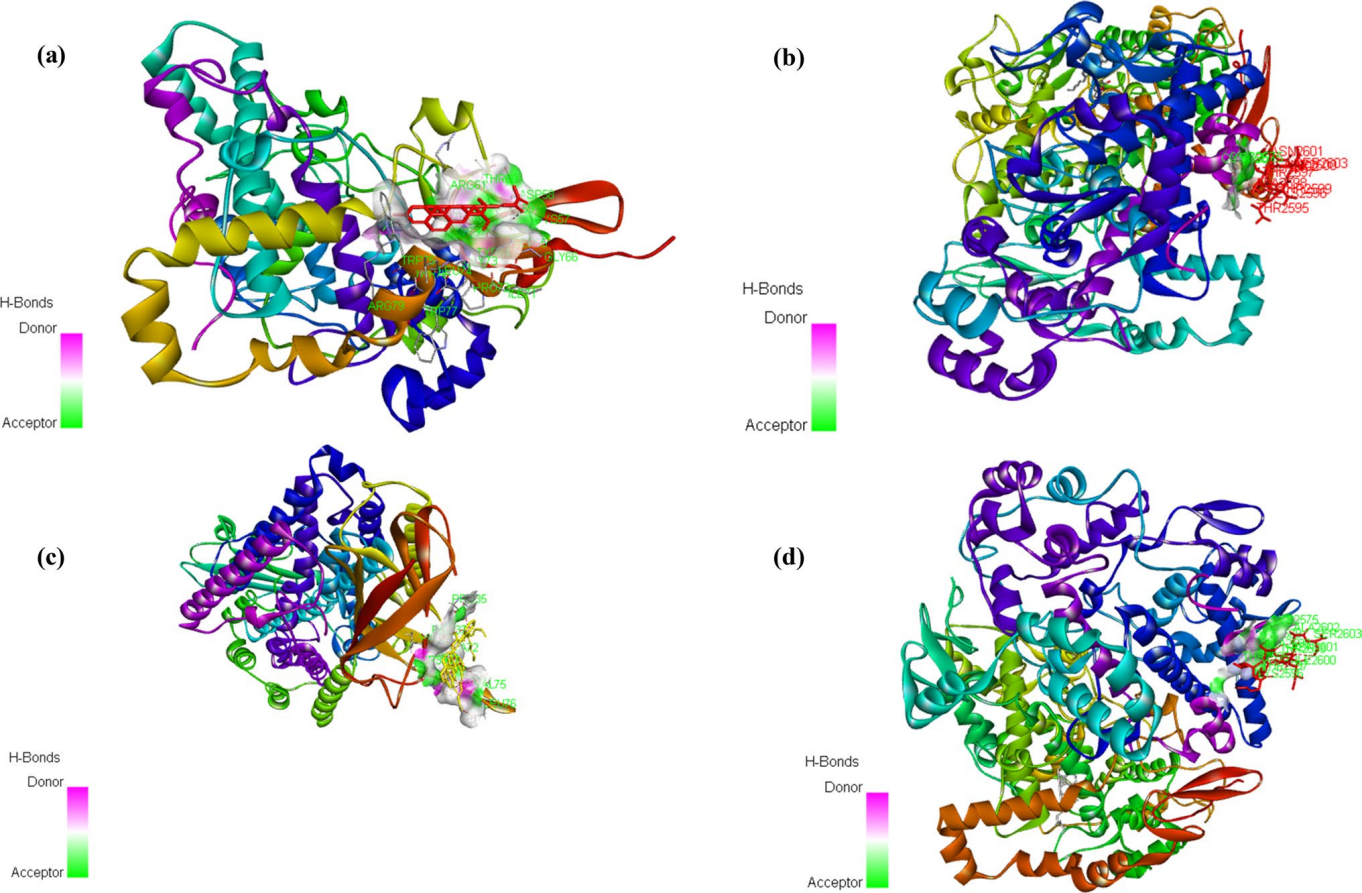
The docked pose and 2D image of Trilinolein (C12) with the active site of **a** COX-1; **b** COX-2; **c** 15-LOX; and **d** Ergosterol (C14) with COX-2

The in silico docking studies determined the inhibitor binding constant Ki and binding energy of the ligand molecules identified in the ethanolic extract of mushroom, *Pleurotus florida* (Tables 4, 5, 6; Figs. 12, 13, 14). The binding energies and Ki of the recognized drug compounds indomethacin for COX 1, Diclofenac for COX-2, and baiclein for 15-LOX were then compared to these results. The ethanolic extract of *Pleurotus florida* included Trilinolein (C12), a ligand molecule which showed the best ability to bind to all three enzymes with more binding energy and Ki values than the corresponding positive controls. Likewise, Ergosterol (C13) could be ranked second in terms of better binding energy towards COX-2 and 15-LOX in comparison to its respective positive controls. Other ligand molecules namely Pentadecanoic acid (C4), hexadecanoic acid (C5), 1(1- Adamantyl)−3-(Dimethylamino)−1-Propanone (C9), Octanoic acid 2-dimethylaminoethyl ester (C10) also showed better binding energy and Ki values for 15-LOX in comparison to the positive control Baicalein. The ideal energy needed for a ligand to bind to its receptor molecule is known as binding energy. The release of binding energy during a drug molecule's interaction with its target results in a decrease in the complex's total energy. The potency of the ligand or inhibitor towards the protein or enzyme is indicated by the inhibition constant (Ki). It is a quantitative measurement that is connected to the concentration at which 50% of an enzyme or protein is inhibited, known as the half-maximal inhibitory concentration [133] implying that the ligand's binding affinity for the enzyme or protein increases with decreasing Ki. Therefore, factors like binding energy and Ki need to be taken into account when assessing a compound's efficacy at an enzyme or protein's active site. The ligand molecule trilinolein exhibited stronger binding affinity, as indicated by a lower binding energy, and a better binding efficiency, as indicated by lower Ki values, according to a comparison of the interaction with positive controls at the binding site. Thus, it could serve as a lead molecule in the creation of new treatments as the Ki values found in the binding experiments authenticated it as an effective potent molecule towards the inhibition of selected pro inflammatory mediators (COX-1, COX-2, 15-LOX).

**Table 4:** Inhibitor binding constant Ki and binding energy of the ligand molecules

COX-1 (6Y3 C)			
S. no	Molecule	Binding energy (B.E) (kcal/mol)	Estimated inhibition constant (EIC) Ki
1	Indomethacin (POSITIVE CONTROL)	− 8.33	778.99 nM
2	C1	− 6.03	38.27 µM
3	C2	− 5.02	268.04 µM
4	C3	− 5.10	183.61 µM
5	C4	− 7.04	6.86 µM
6	C5	− 8.48	1.00 µM
7	C6	− 6.01	39.02 µM
8	C7	− 6.43	19.34 µM
9	C8	− 5.05	197.40 µM
10	C9	− 6.45	18.56 µM
11	C10	− 5.58	81.0 µM
12	C11	− 7.36	4.02 µM
13	C12	− 12.73	464.03 pM
14	C13	− 8.18	1.01 µM

**Table 5:** Inhibitor binding constant Ki and binding energy of the ligand molecules

COX-2 (1 CVU)			
S. no	Molecule	Binding energy (B.E) (kcal/mol)	Estimated inhibition constant (EIC) Ki
1	Diclofenac (POSITIVE CONTROL)	− 8.58	516.65 nM
2	C1	− 6.07	35.83 µM
3	C2	− 5.41	108.68 µM
4	C3	− 5.19	155.61 µM
5	C4	− 7.95	1.49 µM
6	C5	− 8.95	277.15 nM
7	C6	− 6.07	35.44 µM
8	C7	− 7.27	4.68 µM
9	C8	− 5.30	131.14 µM
10	C9	− 7.25	4.81 µM
11	C10	− 6.49	17.47 µM
12	C11	− 7.78	1.98 µM
13	C12	− 13.13	238.98 pM
14	C13	− 12.92	340.29 pM

**Table 6:** Inhibitor binding constant Ki and binding energy of the ligand molecules

15-LOX (4 NRE)			
S. no	Molecule	Binding energy (B.E) (kcal/mol)	Estimated inhibition constant (EIC) Ki
1	Baicalein (Positive Control)	− 7.21	5.16 µM
2	C1	− 5.68	68.13 µM
3	C2	− 5.08	189.51 µM
4	C3	− 4.88	263.11 µM
5	C4	− 7.37	3.99 µM
6	C5	− 7.41	3.71 µM
7	C6	− 5.64	73.03 µM
8	C7	− 7.12	6.06 µM
9	C8	− 4.14	922.36 mM
10	C9	− 7.48	3.29 µM
11	C10	− 7.70	2.29 µM
12	C11	+ 0.40	NIL
13	C12	− 11.25	5.64 nM
14	C13	− 7.76	2.06 µM

**Fig. 15:**
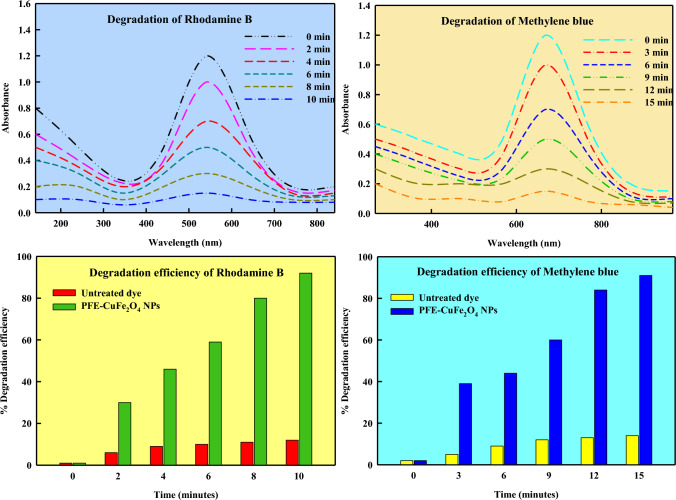
Dye degradation by PFE-CuFe_2_O_4_ NPs

**Fig. 16:**
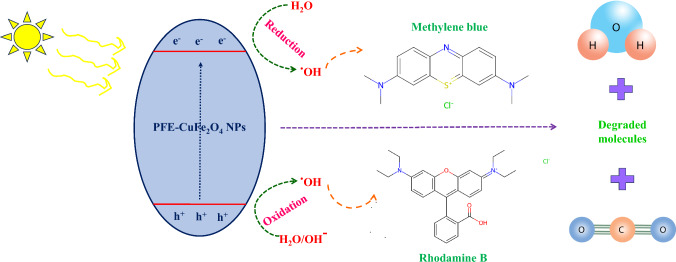
Dye degradation mechanism catalyzed by PFE-CuFe_2_O_4_ NPs

**Fig. 17:**
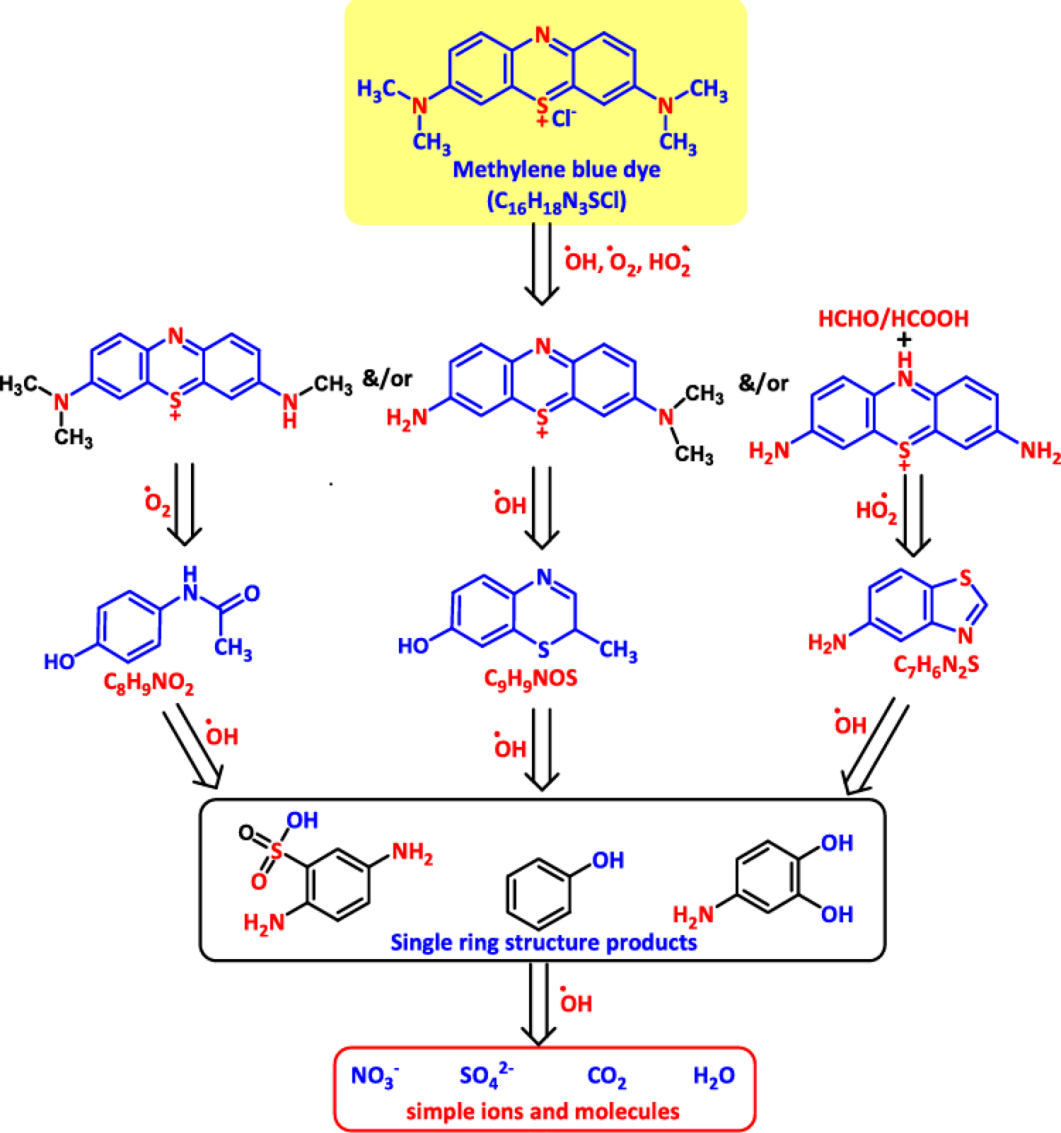
Steps involved in MB dye degradation (Adapted from [150–152])

**Fig. 18:**
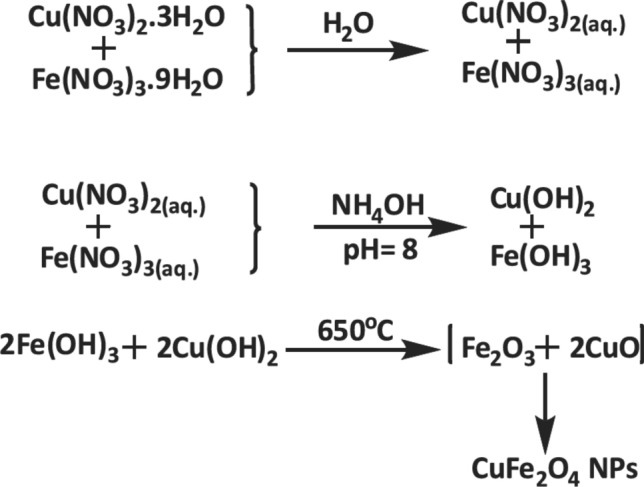
Reactions involved in the formation of CuFe_2_O_4_ NPs (Adapted from Jabeen et al. [27])

The restoration of air, sediment, water, and soil through sophisticated technologies is necessary to address environmental pollution, a global health concern that remains unresolved. This pollution can be caused by inorganic pollutants like metal/metalloids, organic pollutants and harmful dyes [134–136]. Because of the potential harm these pollutants provide to human health, it is necessary to use phytoremediation—plants—or nano-microbioremediation—the use of microorganisms in the presence of nanoparticles. Myco-remediation employs fungi, such as mushrooms, to remediate or biodegrade contaminants through various mechanisms, including biodegradation, bioconversion, bioaccumulation, and biosorption. In addition, mycoremediation is an economical and environmentally responsible way to address the growing problem of land and water pollution [137]. The benefits of myco-remediation over biodegradation include the capacity to oxidize pollutants, low cost and safety, production of a variety of coupling products, effective generation of biodiesel, and tolerance to salinity conditions. The drawbacks enlisted: the need for addition of nutrients, the incapacity to eliminate toxicity, lengthy periods of depletion, and the requirement for immobilization [138]. An investigation of how PFE-CuFe_2_O_4_ NPs degraded dyes Methylene blue (MB) and Rhodamine B (RhB) under direct sunlight was conducted. After the as-prepared reaction mixture was kept under dark and vigorously stirred for 10 min, adsorption–desorption equilibrium was reached. Following 15 min exposure to sunlight, the solution comprises dye molecules that have been adsorbed onto the PFE-CuFe_2_O_4_ NPs catalyst. A UV–vis spectrophotometer was used to track the catalytic activity of the nanomaterial every 2–3 min. The degradation of MB dye was determined to be 91%, and fully degraded after 15 min, as shown in Fig. 15. Similarly, the degradation of RhB dye was determined to be 92%, and fully degraded after 10 min, as shown in Fig. 15. At the beginning of the reaction, the catalyst surface had an additional significant number of occupied active sites. However, as reaction progressed, the active sites decreased due to electron transfer, which caused the compounds to discolor [139]. The discoloration resulted from the breakage of conjugation bonds in the dye molecule [140]. The absorption band of dye methylene blue was found to be at 663 nm, and it may be the result of an electron moving from π − π* or n − π* [141]. After adding the biosynthesized nanocatalyst to the aqueous dye, the absorption band gradually decreases at regular intervals, confirming the nanocatalyst's significant photocatalytic activity through photolysis [142]. According to earlier research demonstrating significant photocatalytic activity, low band gap value, high active surface area, and smaller crystallite size of the particle, are connected with the transfer of energy, creation, and destruction of photo-generated carriers [143]. The solution mixture absorbs photons from the sun (Fig. 16). It releases electrons from the valence band to the conduction band, causing an electron–hole pair to develop in the nanoparticles and causing the dye to discolor generally. The superoxide radical is created when the swinging electrons interacts with molecular oxygen. In this mechanism (Figs. 16, 17), holes formed in the valence band react to produce hydroxyl radicals from H_2_O molecules. Superoxide radicals are created during the cleavage of dye molecules, which speeds up the process. This happens as a result of the surface of the nanoparticles being enhanced with flavonoid and phenolic groups [144]. Moreover, superoxide radicals are transformed into innocuous byproducts like water and carbon dioxide. The rate kinetics of photodegradation process to time under light irradiation was analyzed using the pseudo-first-order kinetic reaction model by employing the following equation:

$$Ln\frac{At}{Ao}= -Kt$$

where A_o_ = initial absorbance of dye, A_t_ = absorbance at time ‘t’, ‘t’ = time taken to absorb dye, ‘K’ = rate constant of first-order reaction.

**Fig. 19:**
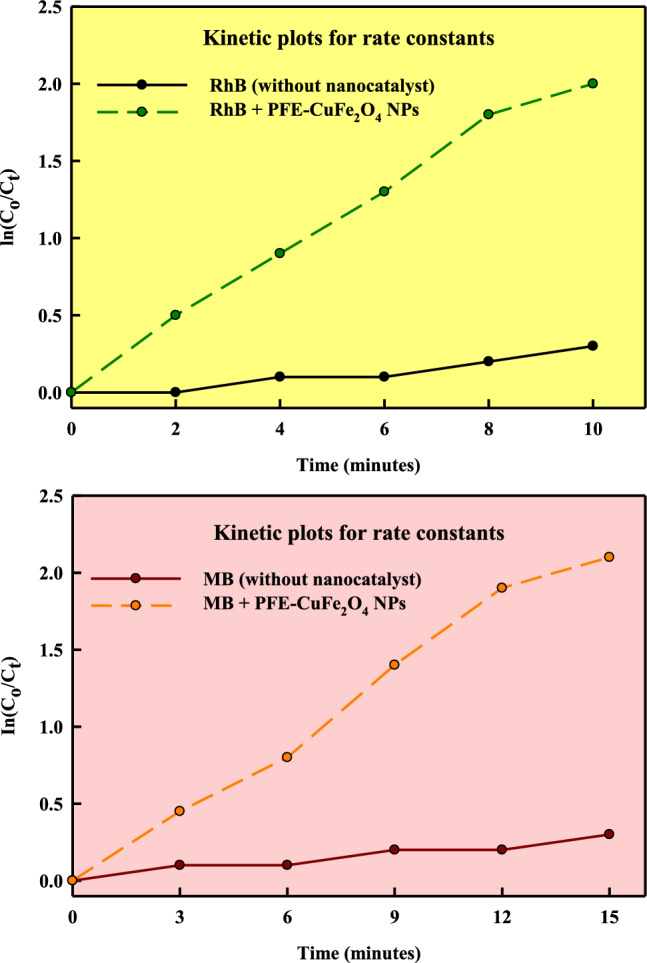
Catalytic degradation of dyes: Kinetic plots for the estimation of rate constants

The data plotted against time"t"yielded a perfect linear fit with high correlation coefficient (R^2^) values of 0.91 and 0.905 for RhB and MB, respectively, which is very close to unity (Fig. 19), suggestive of the degradation reaction exhibiting first-order kinetics. Additionally, in both commercial and biological applications, the toxicity and safety of nanoparticles are essential factors to consider. Because of their unique qualities and larger surface area than larger particles, nanoparticles might behave differently, which raises questions about how they might affect industrial operations, human health, and the environment. Some preventive and toxicological techniques, such as cellular absorption and dissemination, cytotoxicity, biological compatibility, ecological impact, manufacture risk, and regulatory conformance, are considered for industrial and biological applications [145]. Also, the immense hyphal networks of mushrooms are primarily responsible for their benefits, which include strengthening growth by having a metal-binding protein, improving pH and temperature tolerance, improving resistance to complex contaminants, increasing surface area to volume ratio, and producing multifunctional extracellular enzymes [146]. It is important to note that various contaminants from the environment can be mycotoxinated to remove or biodegrade contaminated soil and water through a variety of processes, such as surface sequestration, precipitation, bioconversion, bioaccumulation, biotransformation, biosorption, and biodegradation [147–149].

The present study supports many SDGs (Sustainable Development Goals):

- SDG 3: Exploring the antibacterial characteristics of green synthesized CuFe_2_O_4_NPs to promote health.
- SDG 6: Encouraging the use of possible wastewater treatment solutions to promote sanitation and safe drinking water.
- SDG 9: Formulating CuFe_2_O_4_NPs from natural extracts to promote inventiveness.
- SDG 12: Enhancing ecologically sound organic dye degradation to promote environmentally conscious manufacturing and utilization.
- SDG 13: Promoting effective solar-driven elimination of dyes in the fight against climate change.
- SDG 15: Sustaining ecological balance and biological diversity with eco-friendly CuFe_2_O_4_NPs.
- SDG 17: Building collaborations for multidisciplinary research and innovation centered on the SDGs.

## Conclusion

Green nanoparticle synthesis is becoming increasingly in demand than traditional chemical and physical synthesis, which frequently uses hazardous substances, consumes energy, and harms the environment. Naturally occurring compounds found in fungi and plants have been shown to be viable substitutes for reducing agents in the"green"synthesis process. As they contain a high concentration of bioactive chemicals which can serve as excellent reducers in manufacturing nanoparticles, fungi and mushrooms are particularly fascinating. The present work typifies the green chemistry mediated synthesis of copper ferrite nanoparticles (CuFe_2_O_4_ NPs) using the extract of mushroom *Pleurotus florida* (PFE) as the first-time handiwork. The biofunctionalized CuFe_2_O_4_ NPs were characterized by spectrophotometric studies and electron microscopy reporting spherical shaped CuFe_2_O_4_ NPs with size 22.4 ± 1.4 nm exhibiting absorbance peak at 420 nm. Various organic biochemical compounds assisted in the capping and stabilization of the CuFe_2_O_4_ NPs exemplified by FT-IR with no agglomeration as evidenced by zeta potential value of 28.9 ± 0.2 mV. The GC–MS analysis exemplified 14 compounds in PFE; authenticated by molecular docking studies inferring the presence of Trilinolein, Ergosterol, Pentadecanoic acid, hexadecanoic acid, 1(1- Adamantyl)−3-(Dimethylamino)−1-Propanone, Octanoicacid 2-dimethylaminoethyl ester as molecules of great pharmaceutical potentialities. The biofunctionalized CuFe_2_O_4_ NPs reported pronounced biological functionalities such as antioxidant (77–83%), antibacterial and antibiofilm (84–90%), anti-diabetic (74–80%), and anti-inflammation (43–55%). The PFE functionalized CuFe_2_O_4_ NPs documented 91–92% degradation of dyes MB and RhB exhibiting pseudo first order kinetics with R^2^ values of 0.905 and 0.91, respectively; inferring them as suitable nanocatalysts in water purification and management.

## Supplementary Information

Supplementary Material 1

## Data Availability

Data is provided within the manuscript or supplementary information files.

## Appendix

See Figs. 18, 19.
