# Supplementary material for: Investigation of antibiosis, anti-diabetic, antioxidant, anti-inflammatory, molecular docking and dye degradation potential of green synthesized copper ferrite (CuFe2O4) nanoparticles using mushroom Pleurotus florida
Source: Discov Nano. 2025 Jun 30;20(1):99. doi: 10.1186/s11671-025-04251-5 (PMC12209143; doi:10.1186/s11671-025-04251-5)
Supplement: Supplementary file 1 — Supplementary Material 1 [file 11671_2025_4251_MOESM1_ESM.docx]

**1. α-amylase inhibition assay.** The anti-diabetic potential of CuFe_2_O_4_ NPs was assessed using the α-amylase inhibition assay (Bibi *et al*., 2011). In a sterile microtitre plate, phosphate buffer (15 µl; pH 6.8) and α-amylase enzyme (25 µl; 0.14 U/ml) were added. Following, 40 µl starch solution and 10 µl CuFe_2_O_4_ NPs (concentrations 20–100 µg/ml) were added together, and the mixture was incubated at 50^°^C for 30 minutes. After the incubation period, the mixture was mixed with iodine reagent (90 µl; 5 mM of each potassium iodide and iodine) and 1 M HCl (20 µl). Three reaction controls were taken in parallel: a blank solution without CuFe_2_O_4_ NPs and enzyme, a negative control (solution lacking test sample), and a positive control (acarbose). At 595 nm, the OD values were determined and the following equation (Eq. 1) was used to calculate the % enzyme inhibition:

% Enzyme inhibition = OD(s)-(OD (n))/(OD (b))×100 ------- (Eq. 1)

where OD (b) indicates blank, OD (s) indicates absorbance value of test sample, and OD (n) indicates negative control.

**2. In vitro α‑glucosidase inhibition assay.** The ability of CuFe_2_O_4_ NPs to suppress the activity of the enzyme α-glucosidase was determined using a slightly modified version of Sheliya *et al*. (2016) methodology. The reaction mixture consisted of acarbose, CuFe_2_O_4_ NPs (20-100 μg/ml), and α-glucosidase [7.5 µl; stock solution (0.5 U/ml) in sodium phosphate buffer (pH 6.9; 20 mmol/l)] incubated for 15 minutes at 37^°^C. Additionally, 100μl PNPG (p-nitrophenyl-α-glucopyranoside) was added, and the mixture was incubated for 10 minutes at 37^°^C. At last, the process was bunged by introducing 100μl sodium carbonate (Na_2_CO_3_; 0.1 M). At 405 nm, the absorbance values were measured with acarbose and PNPG + α-glucosidase as the reference and control solutions, respectively.

% inhibition = (control OD-test OD)/(control OD)×100 ------- (Eq. 2)

**3. Antioxidant activities of CuFe_2_O_4_ NPs**

**3.1 FRSA (Free radical scavenging assay).** The efficacy of CuFe_2_O_4_ NPs to scavenge free radicals was examined using the DPPH (2,2-diphenyl 1-picrylhydrazyl) test (Fatima *et al*., 2015). In brief, 190 µL of DPPH reagent and 10 µl CuFe_2_O_4_ NPs were added and incubated for 30 minutes at 37^°^C. The ascorbic acid positive control was run in parallel; the absorbance (OD 515 nm) was measured, and the free radical scavenging activity was assessed using the following equation (Eq. 3):

% Scavenging = 1-AE/AD×100------- (Eq. 3)

where AE= absorbance of test sample solution and AD= negative control.

**3.2 TAC (Total antioxidant capacity).** Using the phosphomolybdenum technique, the total antioxidant activity of CuFe_2_O_4_ NPs was measured (Zafar *et al*., 2016). In summary incubating 900 µl of phosphomolybdenum reagent [sodium phosphate (28 mM), ammonium molybdate (4 mM), and sulphuric acid (0.6 M)] was mixed with 100 µl CuFe_2_O_4_ NPs followed by incubation for 90 minutes at 95^°^C. The absorbance of reaction mixture (200 µl) was measured at 695 nm and the amount of ascorbic acid equivalents to ascorbic acid per mg of test sample (mg AAE/mg) was used to determine the antioxidant activity. Ascorbic acid served as positive control.

**3.3 TRP (Total reducing power).** Using an experiment based on potassium ferricyanide, the overall reducing power of CuFe_2_O_4_ NPs was examined (Zafar *et al*., 2016). The total reducing power was measured using iron as a reducing agent. The reaction mixture, consisting of 40 µl CuFe_2_O_4_ NPs, 1% aqueous potassium ferricyanide, and 400 µl phosphate buffer (pH 6.6, 0.2 mol/l), was incubated for 20 minutes at 45^°^C. Following incubation, aqueous trichloroacetic acid (10%; 400 µl) was added, and the mixture was subjected to centrifugation at 3000 rpm for 10 minutes. The resultant supernatant (500 µl) was combined with the same amounts of sterile distilled water and 100 µl of aqueous FeCl_3_ (0.1%). Following the absorbance measurement at 630 nm, the outcomes were reported as mg AAE/mg. The reaction controls were conducted in parallel using DMSO as the blank and ascorbic acid as the positive control.

**3.4 ABTS antioxidant assay.** The ABTS test was carried out according to Shah *et al*. (2019) guidelines. The mixture containing potassium persulphate (2.5 mM) and ABTS salt (7mM) in equal amounts was stored in the dark for 14–16 hours. The absorbance at 734 nm was measured and normalized to 0.7 before adding CuFe_2_O_4_ NPs. After that, the reaction mixture was mixed with the various concentrations of CuFe_2_O_4_ NPs, and it was left to incubate at room temperature for 15 minutes under dark. The Trolox C equivalent antioxidant capacity (TEAC) was used to quantify the activity after the OD at 734 nm was measured.

**4. Anti-inflammatory potential of CuFe_2_O_4_ NPs**

**4.1 COX-1 and COX-2 inhibitory activities**

COX-1 and COX-2 were used to examine the inhibitory potential of CuFe_2_O_4_ NPs (Jan *et al*., 2021). Ibuprofen (10 mM) and Arachidonic acid (1.1 mM) were taken as positive control and substrate, respectively. Both COXs peroxidase constituents were calculated by following manufacturer's instruction kit. After brief incubation of 5 minutes, the absorbance was read at 590 nm for the quantification of N,N,N/,N/-tetramethyl-p-phenylene diamine.

**4.2 Inhibitory activity of CuFe_2_O_4_ NPs against 15-LOX**

The inhibitory activity of CuFe_2_O_4_ NPs against 15-LOX was examined (Jan *et al*., 2021). Arachidonic acid (10M) and NDGA (100M; nordihydroguaiaretic acid) served as substrate and positive control, respectively. Standard (soy 15-lipooxygenase) was used in the evaluation of hydroperoxides concentration formed as a result of lipo-oxygenation in Tris-HCl buffer (10mM; pH 7.4). The reaction mixture was incubated for 5 minutes and the absorbance was read at 940 nm. Values of absorbance were measured: value A (post 15 min. of substrate incubation) and value B (5 min. inhibitor-enzyme incubation followed by 5-min. chromogen incubation).

**4.3 Inhibitory potential of CuFe_2_O_4_ NPs against secretory phospholipase A2 (sPLA2)**

The assessment of inhibitory activity of CuFe_2_O_4_ NPs against sPLA2 was ascertained by following the method of Jan *et al*. (2021). The compound, thiotheramide-PC (100mM) was taken as substrate and diheptanoyl thio-PC (1.44mM) served as positive control. The free thiols species, released by the breakdown of diheptanoyl thio-PC ester, were quantified by using DTNB (5-50-dithio-bis-butyl) and the absorbance was read at 420 nm. The value of % inhibition was calculated by following formula:

% inhibition=(IA-Inhibitor)/IA×100

**5. Antibacterial activity of CuFe_2_O_4_ NPs**

**5.1 Growth and preservation of pathogenic strains**

*Staphylococcus aureus* and *Escherichia coli* were the two bacterial pathogens that were routinely subcultured on Mueller Hinton Agar and kept as culture stock [10% glycerol stocks (-20^°^C), slants/stabs (4^°^C)].

**5.2 MIC (Minimal Inhibitory Concentration) of CuFe_2_O_4_ NPs**

The broth microdilution method (Baker *et al*., 1991; Cherian *et al*., 2022) was used to determine the MIC. CuFe_2_O_4_ NPs at varying concentrations (20–100µg/ml) were evaluated and kept at 37^°^C for 24 hours with control tubes. At 620 nm, the absorbance values were recorded.

**5.3 MBC (Minimal Bactericidal Concentration) of CuFe_2_O_4_ NPs**

From MIC tubes, aliquots (20 µl) were seeded, cultivated on nutrient agar media, and incubated at 37^°^C for 24 hours in order to determine MBC (Hausdorfer *et al*., 1998; Cherian *et al*., 2020).

**5.4 Antibacterial activity of CuFe_2_O_4_ NPs**

The agar well diffusion method (Magaldi *et al*., 2004; Valgas *et al*., 2007; Cherian *et al*., 2022) was used to measure the bactericidal activity of CuFe_2_O_4_ NPs against pathogens. The 0.1 ml culture (2×10^8^ CFU/ml) was evenly distributed over MHA media. Variable concentrations of CuFe_2_O_4_ NPs (20-100 µg/ml) were added to equal-sized wells, and the latter were incubated for 24 hours at 37^°^C. The ZOI (zone of inhibition) was measured using Antibiotic Zone Scale (HiMedia, India), and cefixime (an antibiotic) served as the positive control.

**5.5 Anti-biofilm potential of CuFe_2_O_4_ NPs**

Crystal violet (CV) assay was used to assess the anti-biofilm efficacy of CuFe_2_O_4_ NPs (Cherian *et al*., 2022). The 96-well microtitre plates were seeded with starter cultures (cell density ~10^8^ cells/ml; 100 µl) cultivated overnight in nutrient broth. Variable concentrations of CuFe_2_O_4_ NPs (20–100 µg/ml; 100 µl) in combination with a control group of untreated cells were added to each well, and the mixture was incubated for 24 hours at 37^°^C. Antibiotic oxacillin was used as control. Three rounds of washings using 1:1 autoclaved distilled water + phosphate buffer saline (PBS) were performed on the residual loosely bound cells. After adding the CV solution (0.25%, 200 µl), the mixture was incubated for 30 minutes at 37^°^C. Autoclaved distilled water mixed with PBS was used to wash the unbound CV. After dissolving the bound CV-bacterial cells in 250 µl of 95% ethyl alcohol, the absorbance at 620 nm was measured.

**6. Molecular docking studies**

The enzyme crystal structures were downloaded from the Protein Data Bank repository, and Table 2 lists the PDB ID for each protein. After the enzymes were downloaded, ligands and/or substrates restricted their active sites. Before docking, the ligands and substrates were removed from the active sites of the enzymes, and the structures were optimized by reducing energy and removing heteroatoms using the Swiss PDB viewer software. The active site that was chosen for the docking studies was validated by comparing the amino acid sequences of the site with the previous data. Through their respective references, the amino acid residues in the active site and binding locations of COX-1 (Miciaccia *et al*., 2021), COX-2 (Kiefer *et al*., 2000), and 15-LOX (Kobe *et al*., 2014) were verified. As they are essential to the enzymes' ability to operate, the cofactors and their connectors were kept within the enzymes. The ChemSketch software was utilized to construct the three-dimensional structures of all the ligands, while the Open Babel GUI software was utilized to convert the structures of the positive controls, which were indomethacin for COX-1 (Tonby *et al*., 2016), diclofenac for COX-2 (Kaur *et al*., 2011), and baiclein for 15-LOX (Ghansenyuy *et al*., 2023) into PDB format. The software Autodock 4.0 was utilized in the docking program to prepare the enzymes by adding Kollman charges and all polar hydrogen atoms. According to Table 1, the binding site for the protein-ligand interaction was located at 60 Å area, which surrounded the active site and had amino acid coordinates. A Lamarckian evolutionary algorithm was used for docking, with a maximum of 2500000 energy assessments and an RMSD tolerance of roughly 2.0A0. Ten simulations were run on the Cygwin 64 terminal. The Root mean square deviation (RMSD) measurements and the minimum binding free energy are the foundation of the analysis. The software Discovery Studio 4.5 Client (http://accelrys.com/products/collaborativescience/biovia-discovery/visualizationdownload.php/) was used to visualize the pose with the best binding affinity. The findings of the ligand binding to COX-1, COX-2, and 15-LOX were contrasted with the corresponding positive controls, indomethacin, diclofenac, and baiclein.

**Table S1.** The PDB ID and binding coordinates of pro inflammatory mediators

| Target protein | PDB ID | Binding site Co-ordinates (X,Y,Z Co-ordinates) |
| --- | --- | --- |
| COX-1 | 6Y3C | X[-10.614]; Y[-47.185], Z[-5.260] |
| COX-2 | 1CVU | X[23.086]; Y[28.833], Z[36.685] |
| 15-LOX | 4NRE | X[-33.223]; Y[-72.156], Z[-26.918] |

Chromatogram of ethanolic extract of *P*. *florida*

**7. Reactive Oxygen Species (ROS) and ETSA Quantification:** The Choi et al. (2006) method was used to determine the amount of reactive oxidative species (ROS) released by the microbes. To conclude, a total of 200 ml of bacterial strain was applied with 1 ml of PFE, CuFe_2_O_4_ NPs, ciprofloxacin (positive control) and DMSO (negative control) and kept in 37^°^C incubator shaker. Once 6 h of incubation had been achieved, the bacteria suspension was centrifuged at 11,000× g for 11 min at low temperature to obtain the pellet. The pellet was applied with 2% Nitro Blue Tetrazolium (NBT) mixture. This pellet was kept at room temperature for 60 min in dark conditions. After centrifugation of the solution, the supernatant was removed, and the pellet was rinsed twice using PBS before another centrifugation at 9000× g for 3 min. The obtained pellet containing cells membrane was disrupted by treating with 2 M KOH solution. A sample of 50% DMSO was combined with the solution and followed by 10 min incubation at room temperature to dissolve formazan crystals. The solution was again centrifuged and 100 μl of the supernatant was distributed to 96 well plates. The absorbance was calculated at 620 nm using ELISA reader. The Electron transport system activity (ETSA) was measured using a previously reported method of Chen et al. (2019).
